# Isolation of a multidrug-resistant *Candida auris* Clade V from an asymptomatic acute myeloid leukemia patient in the intensive care unit

**DOI:** 10.1016/j.mmcr.2026.100794

**Published:** 2026-05-08

**Authors:** Azam Moslemi, Maryam Salimi, Reza Valadan, Zoha Asgari, Javad Javidnia, Tahereh Shokohi, Bram Spruijtenburg, Eelco F.J. Meijer, Mahdi Abastabar

**Affiliations:** a School of Medicine, Mazandaran University of Medical Sciences, Sari, Iran; b Invasive Fungi Research Center, Communicable Diseases Institute, Mazandaran University of Medical Sciences, Sari, Iran; c Molecular and Cell Biology Research Center (MCBRC), Mazandaran University of Medical Sciences, Sari, Iran; d Department of Medical Sciences, Islamic Azad University Sari Branch, Sari, Iran; e Islamic Azad University Sari Branch, Sari, Iran; f Department of Medical Mycology, School of Medicine, Mazandaran University of Medical Sciences, Sari, Iran; g Department of Medical Microbiology, Radboudumc, Nijmegen, the Netherlands; h Radboudumc-CWZ Center of Expertise for Mycology, Nijmegen, the Netherlands; i Department of Medical Microbiology and Immunology, Canisius-Wilhelmina Hospital (CWZ)/Dicoon, Nijmegen, the Netherlands

**Keywords:** *Candida auris*, Acute myeloid leukemia, Antifungal resistance, Whole genome sequencing, Iran

## Abstract

*Candida auris* is a multidrug-resistant fungal pathogen, that rapidly spreads within healthcare settings. Here, an acute myeloid leukemia patient undergoing chemotherapy was admitted to the intensive care unit. From the skin, *C. auris* was isolated and with whole genome sequencing allocated to clade V. Antifungal susceptibility testing showed azole and echinocandin resistance, with the presence of the *ERG11*^*Y132F*^ mutation, while *FKS1* was wild-type. This report presents the first multidrug resistant *C. auris* clade V isolate.

## 1 Introduction

*Candida auris* (also known as *Candidozyma auris*), a yeast belonging to the Ascomycota, is an emerging fungal pathogen that presents significant challenges in healthcare settings because of its multidrug resistance and survival in the hospital environment, causing persistent outbreaks [1]. The emergence of *C. auris*, first reported from Japan in 2009, was initially found to be pan-susceptible to antifungal agents [2]. Since the first report, *C. auris* has disseminated globally and is currently classified by the World Health Organization (WHO) as a critical priority group of fungal pathogen with high mortality rates [3].

*C. auris* can colonize humans and survive on hospital surfaces, facilitating both person-to-person transmission and nosocomial outbreaks [4]. Patients may carry *C. auris* on body location without exhibiting any clinical signs or symptoms. The skin is the most frequent site of colonization, particularly axillae and groin [5]. Prior skin colonization with *C. auris* is a well-recognized risk factor for subsequent invasive disease, with candidemia occurring in up to 25% of critically ill colonized patients in countries with a high *C. auris* prevalence [6]. Both colonized and infected individuals pose a significant risk for transmission, as they can transfer *C. auris* to nearby surfaces and objects, serving as a source of hospital-acquired infections. Infected patients often experience prolonged hospitalization or ICU stays, typically longer than those observed for candidemia caused by other *Candida* species, reflecting the hospital-acquired nature of invasive *C. auris* infections [4]. Adequate diagnostics and infection prevention and control, including the use of approved (sporicidal) disinfectants effective against *C. auris*, are essential to control the spread of this communicable pathogen [1].

Whole-genome sequencing has identified six distinct genetic clades of *C. auris*, each associated with a specific geographic region: South Asia (clade I), East Asia (clade II), South Africa (clade III), South America (clade IV), Iran (clade V), and Indomalaya (clade VI) [7]. The identification of these new clades underscores the importance of ongoing surveillance and genomic analysis to monitor the global distribution and genetic diversity of *C. auris.* Resistance to fluconazole is frequently observed in certain *C. auris* clades and is mainly associated with clade specific point mutations in the *ERG11* gene. Typical substitutions include Y132F and K143R for clade I, VF125AL for clade III, and Y132F and K143R for clade IV, whereas isolates belonging to clades II, V, and VI generally remain susceptible to fluconazole [7,8].

Here, we report the sixth isolation of *C. auris* Clade V in Iran from a patient with acute myeloid leukemia (AML) in an intensive care unit (ICU). Due to its environmental resilience, multi-drug resistance, and high mortality rate, every *C. auris* case constitutes a critical threat to patient safety.

## 2 Case presentation

On August 23, 2024, as part of an ongoing study to identify colonizing *Candida* species in hospitalized patients, skin swabs were collected from a 51-year-old male with (AML) in the intensive care unit (ICU) of Imam Khomeini Hospital, Sari, Iran. Patient history included induction chemotherapy with daunorubicin and cytarabine in the months before admission. After the induction, he had been admitted twice for profound cytopenias and generalized weakness. Presently, he presented with profound weakness, fatigue, and fever. In clinical assessment he had no significant cardiopulmonary abnormalities. Laboratory investigations revealed a hemoglobin level of 8.9 g/dL, hematocrit of 26%, white blood cell count of 39,200/μL with approximately 68% blasts on the peripheral smear, and platelet count of 45,000/μL. Serum LDH and uric acid levels were elevated to 982 U/L and 8.8 mg/dL, respectively. A procalcitonin level of 0.12 ng/mL was measured, arguing against a diagnosis of bacterial sepsis. Based on the clinical and laboratory findings, refractory AML was diagnosed and the patient was admitted to the ICU for further evaluation and chemotherapy.

**Fig. 1:**
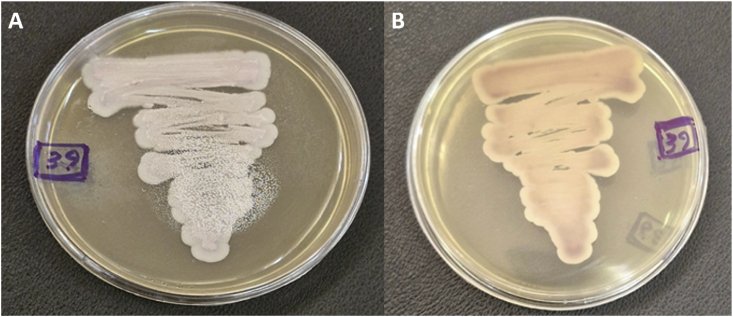
*Candida auris* colonies on CHROMagar Candida (pink color) after 48 hours incubation 30 °C: (A) obverse; (B) reverse.’

**Fig. 2:**
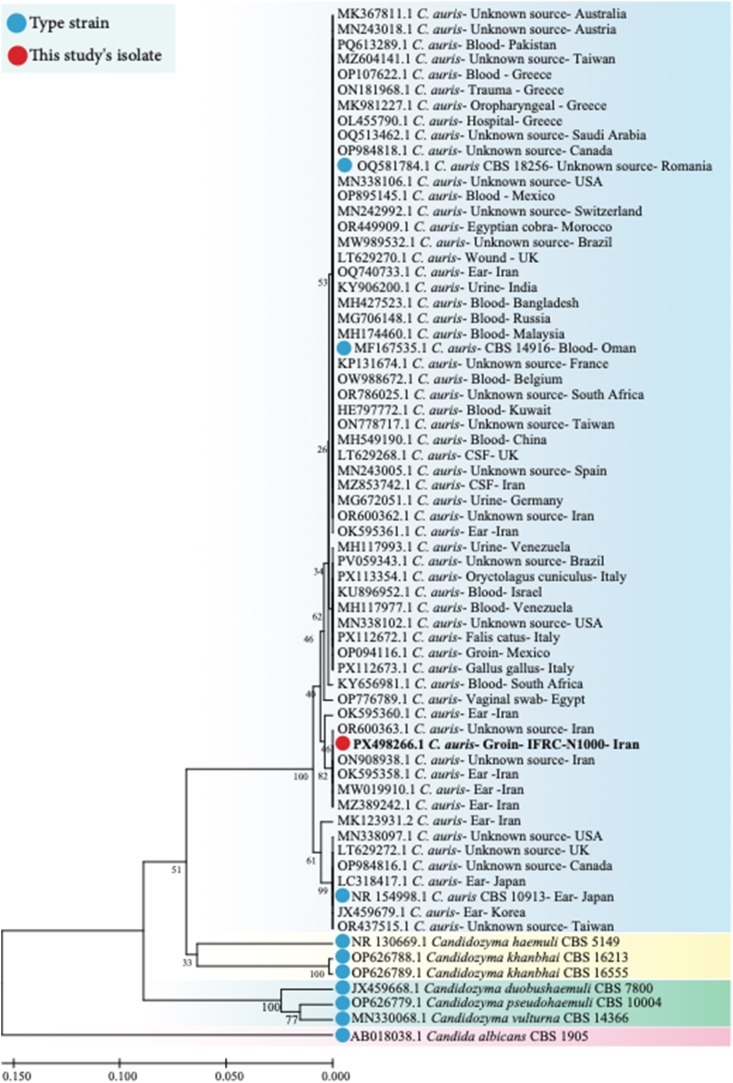
Phylogenetic tree generated by MLH analysis using ITS sequences of *Candida auris* strains with closely related *Candida* species. Bootstrap support values above 70% are indicated at the nodes. Red dot indicates *C. auris* strain identified in this study (GenBank accession number PX498266).

The skin swabs were inoculated on Sabouraud dextrose agar (Difco, USA) supplemented with chloramphenicol and on CHROMagar Candida (CHROMagar Company, France) and incubated at 30 °C and 42 °C for 48 hours. After culture, direct microscopic examination revealed round to ovoid yeast cells with colonies displaying pale pink to dark purple coloration on CHROMagar Candida, suggesting a non-*albicans Candida* species (Fig. 1). Notably, the isolate was also able to grow at 42 °C, a characteristic feature supporting its identification as *C. auris*. For accurate species identification, the isolate was characterized by amplifying the internal transcribed spacer (ITS) region with ITS1 and ITS4 primers as per established protocols [9]. Comparative analysis of the sequence data with reference sequences in the NCBI database was done using nucleotide blast (https://blast.ncbi.nlm.nih.gov/Blast.cgi) The isolate has been deposited in the reference culture collection of the Invasive Fungi Research Center (IFRC) with the accession number IFRCN1000. Additionally, the obtained nucleotide sequences have been submitted to GenBank under the accession number PX498266. A phylogenetic tree was constructed using the neighbor-joining method in MEGA X (v10.1), with branch support assessed through 1000 bootstrap replicates. ITS sequences demonstrated 100% similarity with multiple *C. auris* isolate from Iran which were allocated to clade V (Fig. 2).

Antifungal susceptibility testing (AFST) was performed following the Clinical and Laboratory Standards Institute (CLSI) M27 4th edition guideline [10]. Fresh cultures were diluted to 1 × 10^3^ CFU/mL in RPMI-1640 medium. Minimum inhibitory concentrations (MICs) were read visually after 24 hours of incubation at 35 °C as the lowest antifungal concentration with a 50% growth reduction as compared to the growth control, except amphotericin B for which complete growth reduction was used. CLSI broth microdilution testing yielded the following MICs: fluconazole ≥64 μg/mL; itraconazole, anidulafungin, caspofungin, miconazole, and clotrimazole at ≥16 μg/mL; and voriconazole and amphotericin B at 1 μg/mL.

**Fig. 3:**
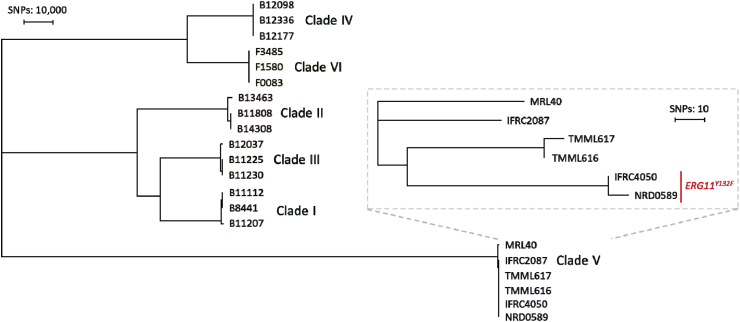
Whole genome sequencing single nucleotide polymorphism tree of the *Candida auris* isolate recovered from the current patient (NRD0589).

WGS was performed on the Illumina NovaSeq 6000 platform (Illumina, San Diego, CA, USA) at Eurofins Genomics (Ebersberg, Germany). Genomic libraries were sequenced in a 2 × 150 bp paired-end read mode. Read data were sourced from the SRA database and processed using a previously established pipeline for alignment and variant calling, using the B11220 reference genome (GCA_003013715.2) [11]. With Nucleotide Blast the *ERG11* (OL742093.1), *ERG3* (OK564587.1)*, ERG6* (OK564623.1)*, TAC1b* (OL742107.1), *ERG3* (OK564587.1), *CIS2* (XM_029035297.2) and *FKS1* (OQ632644.1) genes were located in the B11220 reference genome and visualized with IGV. All raw read data generated in this study have been deposited in the NCBI SRA database under BioProject accession number PRJNA1381094. Fluconazole resistance correlated with the presence of the *ERG11*^*Y132F*^ mutation, while no mutations were found in *FKS1*, *ERG3* and *CIS2*. Using WGS SNP analysis, the isolate was allocated to clade V and found to be closely related to IFRC4050 with a difference of 11 SNPs, which also harbors the same mutation (Fig. 3).

## 3 Discussion

The present report describes the sixth clade V *C. auris* case in Iran from a patient with AML in the ICU without active infection. This finding highlights the critical role of active screening and microbiological surveillance in high-risk hospitalized patients, especially those with hematological malignancies undergoing chemotherapy, radiotherapy, or with prolonged neutropenia.

The patient in this study had several predisposing risk factors, including underlying malignancy, prolonged ICU stay, chemotherapy, and possibly a history of broad-spectrum antibiotics [12]. Although the patient had no clinical symptoms of invasive fungal infection, cutaneous colonization with *C. auris* was detected. This colonized state is a known potential reservoir for nosocomial transmission of this pathogen and can lead to nosocomial outbreaks, especially in sensitive settings such as ICUs and hematology oncology wards [12].

Molecular identification and sequencing of the ITS region allocated this isolate to clade V (Iranian clade). Phylogenetic analysis showed clear clustering of this isolate with other Iranian isolates, suggesting a common genetic origin and possibly an endemic rotation of this clade within the region, which was subsequently confirmed by WGS. Of particular note in this report is the identification of a Y132F point mutation in the *ERG11* gene in this isolate. Although this mutation is typically associated with fluconazole resistance in clade I (South Asia), we report the second observation in clade V, which is generally considered to be susceptible to fluconazole [13]. Given that all clade V isolates form a monophyletic branch, they likely originate from a shared common ancestor that already acquired this mutation and subsequently spread. This finding underscores the paramount importance of genomic surveillance of *C. auris* isolates in Iran to track the emergence and spread of new resistance mechanisms and understand the population dynamics of this pathogen.

The present isolate displayed very high MICs to fluconazole compared to the isolate reported by Abastabar et al., in 2019 from a patient with otomycosis [14]. Although that isolate was also resistant to fluconazole (MIC = 16 μg/mL), its MIC using the same methodology was considerably lower. In contrast to our isolate, it remained susceptible to voriconazole and itraconazole. The strongly elevated azole and echinocandin MICs in this isolate suggests that additional resistance mechanisms are involved for the current isolate, although inspection of resistance-associated genes were unable to uncover this. Although the majority of echinocandin resistant *C. auris* isolates harbors *FKS1* mutations, other reported mechanisms involve mutations in *ERG3* and *CIS2* [15], which were also not present in this isolate. These phenotypic differences may reflect further adaptation to antimicrobial selection pressures in different clinical settings.

Including the current report, a total of eight Iranian *C. auris* cases are reported to date, of which six belong to clade V and two to clade I [13,16,17]. One of the clade I isolates also showed resistance to fluconazole (MIC ≥32 μg/mL) with the same *ERG11* mutation while the other was like most clade V isolates pan-susceptible. Thus, Iran is currently facing the challenge of dealing with the coexistence of two different genetic clades (I and V) of this pathogen, each of which may have its own resistance and prevalence patterns. These findings further emphasize the urgency of establishing an active surveillance system, screening patients with a history of healthcare abroad, and strengthening international coordination to track and contain this pervasive public health threat.

In conclusion, the present report describes the isolation of a multidrug-resistant isolate of *C. auris* from the Iranian endemic clade V from a patient with asymptomatic acute myeloid leukemia. The identification of the Y132F mutation in the *ERG11* gene in this isolate, and the first report of echinocandin resistance in clade V, are an alarming sign of the emergence of resistant strains in the fungal population within the country. These findings clearly indicate that *C. auris* is a real and present threat in Iranian hospital settings. Therefore, implementation of active surveillance programs, accurate molecular diagnostics, resistance profiling, and strict adherence to infection control protocols are urgently needed to prevent future outbreaks and maintain patient safety.

## CRediT authorship contribution statement

**Azam Moslemi:** Writing – original draft, Methodology, Investigation, Formal analysis. **Maryam Salimi:** Methodology, Investigation, Formal analysis. **Reza Valadan:** Data curation, Investigation, Writing – review & editing. **Zoha Asgari:** Data curation, Writing – review & editing. **Javad Javidnia:** Data curation, Writing – review & editing. **Tahereh Shokohi:** Writing – review & editing, Supervision, Conceptualization. **Bram Spruijtenburg:** Writing – review & editing, Methodology, Formal analysis. **Eelco F.J. Meijer:** Writing – review & editing, Funding acquisition. **Mahdi Abastabar:** Writing – original draft, Supervision, Funding acquisition, Conceptualization.

## Ethical statement

This study was approved by the Ethics Committee of Mazandaran University of Medical Sciences (IR.MAZUMS.REC.1402.365, september 13, 2023) and adheres to the Declaration of Helsinki. Written consent was obtained from the patient for publication of this case report.

## Conflict of interest

Eelco F.J. Meijer received research grants from Mundipharma and Scynexis, is in the scientific advisory board for Pfizer and has received speaker fees from Gilead Sciences. All other authors declare no conflicts of interest.
